# Right inferior frontal cortex: addressing the rebuttals

**DOI:** 10.3389/fnhum.2014.00905

**Published:** 2014-11-11

**Authors:** Adam R. Aron, Trevor W. Robbins, Russell A. Poldrack

**Affiliations:** ^1^Department of Psychology, University of California at San DiegoLa Jolla, CA, USA; ^2^Department of Psychology and Behavioural and Clinical Neuroscience Institute, University of CambridgeCambridge, UK; ^3^Department of Psychology, Stanford UniversityPalo Alto, CA, USA

**Keywords:** response inhibition, inhibitory control, right inferior frontal gyrus, monitoring, lesions

We recently provided an updated theory of the role of posterior ventral right inferior frontal cortex (hereafter rIFC) in inhibitory response control (Aron et al., 2014). We claimed that when rIFC is triggered by a stop signal, unexpected event or endogenous rule, it engages a brake; i.e., it slows, pauses, or completely stops an action via one or more rIFC-based fronto-basal-ganglia networks.

This account was challenged by two recent papers: “Ten years of inhibition revisited” by Swick and Chatham (2014), and “A functional network perspective on response inhibition and attentional control” by Erika-Florence et al. (2014). Here we address the points made in those papers.

Swick and Chatham (hereafter S&C) argue that rIFC instead monitors for cues that signal a change of action, and question our interpretation of Chatham et al. (2012). In that study, for the stop signal task, subjects tried to stop when a signal occurred; in the double Go task, when the signal occurred, subjects continued their response and then repeated it. Right IFC was activated both for stop and double Go trials. However, the signal on double Go trials slowed the primary response, consistent with infrequent events recruiting a brake. S&C further showed that double Go trials without primary task slowing also activated the rIFC. But such activation could still reflect a brake that fails to slow the response. S&C ask: (a) *Why should braking occur on such trials when they are contrary to task goals (to respond quickly)*? We propose, along with others (Chatham et al., 2012; Wiecki and Frank, 2013), that braking, at least of the externally triggered, rather than endogenously triggered variety, is inextricably linked with salience detection—at least when salient signals are relevant to ongoing behavior (also see Wessel and Aron, 2014). (b) *How can we argue that the above rIFC activation reflected “braking” when it was too late to affect behavior, when, at the same time, it has been shown that rIFC BOLD correlates with SSRT* (Whelan et al., 2012)? We see no contradiction here. Right IFC activation *within a subject* could be similar for double Go trials (slowed or not) and stop trials (successful or not)—reflecting triggering of rIFC. On the other hand, rIFC activation *between-subjects* varies for successful stop trials, with several studies showing increased activation for faster stop speeds (Aron and Poldrack, 2006; Congdon et al., 2010; Whelan et al., 2012; Cai et al., 2014). (c) *Why is rIFC more strongly recruited on double Go trials than during the stop task itself?* That comparison is confounded by the double Go condition always being performed first. Activation decreases with practice (Kelly and Garavan, 2005) although within-session practice effects specifically for response inhibition paradigms have not, to our knowledge, been reported. (d) *Why is rIFC recruitment sustained even when subjects must always produce a “go” response and proactive inhibitory control is unnecessary?* Activation for the contrast of double Go vs. rest blocks covered almost the entire right frontal lobe, making interpretation difficult in relation to braking.

Using ECoG we showed rIFC activation even when a stop signal did not occur (but was merely expected), and that the time of activation was around the response and not the anticipated time of the stop signal (Swann et al., 2013). S&C correctly observe that the tight timing to the response only occurred in 2 out of 5 patients. However, we then showed (Wessel et al., 2013) that rIFC direct electrical stimulation (DES) just before the response induced slowing in a braking context. S&C argue this could arise from non-specific effects of DES (mentioning that frontal DES at 60 Hz induces visual hallucinations). But note: (i) our frontal effect occurred relative to a control site, using two brief pulses of DES (not the 60 Hz method, nor the “short train” method of 5 pulses, with 3 ms interpulse interval, Axelson et al., 2009) (ii) it was context-specific—slowing only those subjects who were not already slowing, (iii) it more strongly affected trials on which braking was needed, and (iv) DES like ours *can* have highly specific effects (Desmurget et al., 2013). It could be argued that such rIFC recruitment reflects a kind of monitor (Chatham et al., 2012; Chevalier et al., 2014) that activates in proximity to the irrevocable/ballistic stage of an action, so long as that action is contingent on the absence of some countermanding signal. However, it is unclear how disrupting the activity of this putative monitor would elongate RT unless response initiation has to wait until the monitor function is complete; yet other work has shown that responses are initiated as early as 200 ms after the go stimulus (Jahfari et al., 2010). For us, the data are most compatible with rIFC being part of a brake.

S&C question the anatomical specificity of our ECoG studies to rIFC. While our supplementary data (Swann et al., 2009) showed the successful vs. failed stop increase most consistently within rIFC, it did also occur in premotor cortex. Our subsequent reports also showed the power increase in rIFC (Swann et al., 2012; Wessel et al., 2013) but we did not analyze all the electrodes as these studies were hypothesis-driven. We will eventually provide a fuller picture.

S&C say we ignored the study of Verbruggen et al. (2010), in which TMS of rIFC prolonged SSRT but also the making of a second response. That study proposed that rIFC updates action plans in general: in a stop context it “brakes” the response, and in a dual response context it reprograms an alternative action. Although reprogramming could involve first applying a brake, if it were shown that it is purely reprogramming then we would accept a more general rIFC role. However, an alternative is the likely spatial spread of TMS over wider posterior rIFC, possibly affecting both putative “braking” in rIFC and reprogramming in adjacent ventral premotor cortex (Buch et al., 2010).

We now turn to the challenge by Erika-Florence et al. (2014) (hereafter EF et al.). This fMRI study included a stop task and several putative non-inhibitory tasks. From right lateral PFC and insula, 7 ROIs were generated from independent components analysis. Data in several ROIs showed transient activation during the stop task, but also for the other tasks leading to the conclusion that there is no “prefrontal inhibitory module.” However, we nowhere claimed such a module. In our first review (Aron et al., 2004) the full sentence (“Whereas neuroimaging implicates diverse PFC foci, advances in human lesion-mapping support the functional localization of such inhibition to right IFC alone.”) makes clear that we were specifically claiming that rIFC was alone amongst *prefrontal* regions in implementing inhibition. We also anticipated involvement of a broader network (“Future research should investigate … [rIFC] interaction within a wider network”), which is now well ratified. Second, the EF study was not focused on the crucial posterior ventral rIFC region. While we did confirm, in data kindly provided by the authors, that components 2, 4, 6, and 7 had activation overlapping with rIFC (i.e., MNI 48 16 18 from meta-analysis, Levy and Wagner, 2011), consistent with braking in one or more of the tasks, *none* of the small 5 mm spherical ROIs was placed there. EF acknowledged their study may have focused on the “wrong set of IFC subregions” but they argued “… several of these ROIs were related to inhibitory task performance as gauged by correlations between SSRT and functional connectivity measures.” But just because SSRT correlated with some functional connectivity measures between IFC subregions and other regions does not prove that those IFC subregions implement braking. While many hypothesis-driven studies have found correlations between nodes or connections of the putative braking network and SSRT (probably corresponding to the variability in SSRT that is related to braking), SSRT also reflects other sensory and attentional processes (Boucher et al., 2007).

We also have concerns about EF's “non-inhibitory” tasks, e.g., the “respond task.” When the occasional signals occurred, subjects responded according to the direction of the earlier Go arrow on that trial. As RT was fast (~450 ms) subjects were probably always preparing a movement and keeping it in abeyance with a “hold-your-horses” (Wiecki and Frank, 2013) or other (Duque and Ivry, 2009) braking process that could activate rIFC.

EF et al. made much of successful and failed stop trials activating equally. Yet this is long-established by well-powered fMRI analysis (Congdon et al., 2010), probably reflecting that rIFC activation corresponds to incoming triggers to “turn on” the brake, regardless of behavioral success.

Finally, EF's view that rIFC and its networks implement a “general class of attentional and working memory maintenance processes” does not provide easily testable hypotheses and fails to specify how this processing is converted into action.

Turning back to S&C. They raised questions about lateralization. In a substantial sample of patients with right vs. left lateral frontal lesions (Aron et al., 2003), we showed that rIFC but not left lateral damage affected SSRT. But Swick et al. (2008) showed that left lateral frontal damage increased commission errors for a Go/NoGo task. We observed this also occurred for the 50/50 condition where decision-making rather than response inhibition probably occurs. S&C replied that NoGo ability was disproportionately impaired in the 90/10 condition. Yet this could have arisen because patients responded more quickly on Go trials in the 90/10 than 50/50 condition, which could exacerbate a decision-making deficit. To be clear, the task required NoGoing to the letter “X” and not others; this will require, in the 90/10 condition a process of object recognition, rule matching with verbal working memory followed by triggering of a brake; we propose that left IFC damage could have impaired the verbal WM process rather than the brake. The speed pressure of the 90/10 condition could add “noise” the verbal WM process, thus exacerbating the deficit.

S&C claimed we omitted reference to Kramer et al. (2013) which purported to show left IFC to be critical for inhibitory control whilst challenging the critical role of right IFC. This lesion study comprised a small sample of patients with left or right frontal stroke. On a NoGo task, the patients (both hemispheres grouped, and not with specific damage to IFC) made more commission errors than controls, especially for 80/20 vs. 50/50 conditions. This hardly shows that left IFC is critical for inhibitory control. As above, commission errors may reflect decision-making failure (esp. in the 50/50 Go/NoGo condition). Regarding the stop task, only two out of 9 left frontals had a deficit relative to controls (and these left frontals had damage to many regions). In the right frontals, 1 of the 5 had an SSRT deficit compared to controls; and, admittedly three patients, who did have rIFC damage, did not. Thus, whilst right IFC damage sometimes fails to produce a deficit we note it is difficult to interpret null results in chronic lesion studies because of possible re-organization.

A lesion study by Molenberghs et al. (2009) is also relevant. Patients performed a Sustained Attention to Response Task (SART), responding quickly to any digit 1–9 except “3,” which required action withholding. Failing to do so was a commission error. Four out of 19 left frontals had left IFC damage, with commission errors similar to controls. Six out of 25 right frontals had a lesion overlapping with rIFC. Four of these had massively increased commission errors (60% or more), but no difference in omissions or RT compared to controls. The authors restudied two of these patients years later. They replicated the effect of very elevated commission errors using the standard SART, and then showed no deficit at all when the patients were required to Go rather than NoGo to the infrequent signals. This again suggests rIFC is critical for braking but not monitoring, which could relate to a different sub-region, perhaps inferior frontal junction (Levy and Wagner, 2011). Notably a recent meta-analysis purports to dissociate the braking function to rIFC and the stop signal detecting attentional function to the right insula (Cai et al., 2014).

Regarding the locus of braking to rIFC rather than OFC, DLPFC, or left IFC, we suggested this could be falsified by studies that selectively disrupt these regions without damaging connections. S&C ask how could one prove a lack of damage to “connections” in a real frontal patient or TMS subject? To give an example: such a falsifying study could show that damage to rDLPFC and not rIFC impaired stopping (not merely by disrupting rule knowledge), and moreover, using DTI (Mah et al., 2014), that the DLPFC lesion spared connections between rIFC and preSMA.

Regarding the function of rIFC, we argued it does not merely reflect monitoring and that “Our position could be falsified by, for example, a disruption study that (a) uses a task that clearly does require braking, (b) shows that disruption *does not* affect outright stopping or braking/pausing, (c) shows that disruption *does* affect some other function such as attentional detection or monitoring.” S&C ask how could one prove that a task (of monitoring) lacks all inhibitory demands. We propose a test of simple oddball detection, as above (Molenberghs et al., 2009).

Our view, sharpened by this challenging analysis of S&C and EF, is that rIFC is turned on by signals that occur during impending behavior (it can also perhaps be turned on endogenously and tonically). When rIFC is turned on, the brake is engaged. Whether this affects behavior depends on the timing of the signal in relation to the impending behavior (and fMRI of rIFC is not apparently sensitive to whether behavior is affected). Triggering the brake requires a monitor, but that is possibly implemented in a different PFC sector. Higher resolution ECoG and MEG could be useful for understanding the relative timing of PFC subregions for different putative functions.

## Conflict of interest statement

The authors declare that the research was conducted in the absence of any commercial or financial relationships that could be construed as a potential conflict of interest.

## References

[B1] Aron A. R. Fletcher P. C. Bullmore E. T. Sahakian B. J. Robbins T. W. (2003). Stop-signal inhibition disrupted by damage to right inferior frontal gyrus in humans. Nat. Neurosci. 6, 115–116. 10.1038/nn1003 12536210

[B2] Aron A. R. Poldrack R. A. (2006). Cortical and subcortical contributions to Stop signal response inhibition: role of the subthalamic nucleus. J. Neurosci. 26, 2424–2433. 10.1523/JNEUROSCI.4682-05.2006 16510720 PMC6793670

[B3] Aron A. R. Robbins T. W. Poldrack R. A. (2004). Inhibition and the right inferior frontal cortex. Trends Cogn. Sci. 8, 170–177. 10.1016/j.tics.2004.02.010 15050513

[B4] Aron A. R. Robbins T. W. Poldrack R. A. (2014). Inhibition and the right inferior frontal cortex: one decade on. Trends Cogn. Sci. 18, 177–185. 10.1016/j.tics.2013.12.003 24440116

[B5] Axelson H. W. Hesselager G. Flink R. (2009). Successful localization of the Broca area with short-train pulses instead of “Penfield” stimulation. Seizure 18, 374–375. 10.1016/j.seizure.2009.01.005 19201212

[B6] Boucher L. Palmeri T. J. Logan G. D. Schall J. D. (2007). Inhibitory control in mind and brain: an interactive race model of countermanding saccades. Psychol. Rev. 114, 376–397. 10.1037/0033-295X.114.2.376 17500631

[B7] Buch E. R. Mars R. B. Boorman E. D. Rushworth M. F. S. (2010). A network centered on ventral premotor cortex exerts both facilitatory and inhibitory control over primary motor cortex during action reprogramming. J. Neurosci. 30, 1395–1401. 10.1523/JNEUROSCI.4882-09.2010 20107065 PMC2880444

[B8] Cai W. Ryali S. Chen T. J. Li C. S. Menon V. (2014). Dissociable roles of right inferior frontal cortex and anterior insula in inhibitory control: evidence from intrinsic and task-related functional parcellation, connectivity and response profile analyses across multiple datasets. J. Neurosci. 34, 14652–14667. 10.1523/JNEUROSCI.3048-14.2014 25355218 PMC4212065

[B9] Chatham C. H. Claus E. D. Kim A. Curran T. Banich M. T. Munakata Y. (2012). Cognitive control reflects context monitoring, not motoric stopping, in response inhibition. PLoS ONE 7:e31546. 10.1371/journal.pone.0031546 22384038 PMC3288048

[B10] Chevalier N. Chatham C. H. Munakata Y. (2014). The practice of going helps children to stop: the importance of context monitoring in inhibitory control. J. Exp. Psychol. Gen. 143, 959–965. 10.1037/a0035868 24512561 PMC4057641

[B11] Congdon E. Mumford J. A. Cohen J. R. Galvan A. Aron A. R. Xue G.. (2010). Engagement of large-scale networks is related to individual differences in inhibitory control. Neuroimage 53, 653–663. 10.1016/j.neuroimage.2010.06.062 20600962 PMC2930099

[B12] Desmurget M. Song Z. Mottolese C. Sirigu A. (2013). Re-establishing the merits of electrical brain stimulation. Trends Cogn. Sci. 17, 442–449. 10.1016/j.tics.2013.07.002 23932195

[B13] Duque J. Ivry R. B. (2009). Role of corticospinal suppression during motor preparation. Cereb. Cortex 19, 2013–2024. 10.1093/cercor/bhn230 19126798 PMC2722425

[B14] Erika-Florence M. Leech R. Hampshire A. (2014). A functional network perspective on response inhibition and attentional control. Nat. Commun. 5:4073. 10.1038/ncomms5073 24905116 PMC4059922

[B15] Jahfari S. Stinear C. M. Claffey M. Verbruggen F. Aron A. R. (2010). Responding with restraint: what are the neurocognitive mechanisms? J. Cogn. Neurosci. 22, 1479–1492. 10.1162/jocn.2009.21307 19583473 PMC2952035

[B16] Kelly A. M. Garavan H. (2005). Human functional neuroimaging of brain changes associated with practice. Cereb. Cortex 15, 1089–1102. 10.1093/cercor/bhi005 15616134

[B17] Kramer U. M. Solbakk A. K. Funderud I. Lovstad M. Endestad T. Knight R. T. (2013). The role of the lateral prefrontal cortex in inhibitory motor control. Cortex 49, 837–849. 10.1016/j.cortex.2012.05.003 22699024 PMC3443703

[B18] Levy B. J. Wagner A. D. (2011). Cognitive control and right ventrolateral prefrontal cortex: reflexive reorienting, motor inhibition, and action updating. Ann. N.Y. Acad. Sci. 1224, 40–62. 10.1111/j.1749-6632.2011.05958.x 21486295 PMC3079823

[B19] Mah Y. H. Husain M. Rees G. Nachev P. (2014). Human brain lesion-deficit inference remapped. Brain 137(Pt 9), 2522–2531. 10.1093/brain/awu164 24974384 PMC4132645

[B20] Molenberghs P. Gillebert C. R. Schoofs H. Dupont P. Peeters R. Vandenberghe R. (2009). Lesion neuroanatomy of the sustained attention to response task. Neuropsychologia 47, 2866–2875. 10.1016/j.neuropsychologia.2009.06.012 19545580

[B20a] Swick D. Chatham C. H. (2014). Ten years of inhibition revisited. Front. Hum. Neurosci. 8:329. 10.3389/fnhum.2014.00329 24904369 PMC4033166

[B21] Swann N. Tandon N. Canolty R. Ellmore T. M. Mcevoy L. K. Dreyer S.. (2009). Intracranial EEG reveals a time- and frequency-specific role for the right inferior frontal gyrus and primary motor cortex in stopping initiated responses. J. Neurosci. 29, 12675–12685. 10.1523/JNEUROSCI.3359-09.2009 19812342 PMC2801605

[B22] Swann N. C. Cai W. Conner C. R. Pieters T. A. Claffey M. P. George J. S.. (2012). Roles for the pre-supplementary motor area and the right inferior frontal gyrus in stopping action: electrophysiological responses and functional and structural connectivity. Neuroimage 59, 2860–2870. 10.1016/j.neuroimage.2011.09.049 21979383 PMC3322194

[B23] Swann N. C. Tandon N. Pieters T. A. Aron A. R. (2013). Intracranial electroencephalography reveals different temporal profiles for dorsal- and ventro-lateral prefrontal cortex in preparing to stop action. Cereb. Cortex 23, 2479–2488. 10.1093/cercor/bhs245 22879352 PMC3767964

[B24] Swick D. Ashley V. Turken A. U. (2008). Left inferior frontal gyrus is critical for response inhibition. BMC Neurosci. 9:102. 10.1186/1471-2202-9-102 18939997 PMC2588614

[B25] Verbruggen F. Aron A. R. Stevens M. A. Chambers C. D. (2010). Theta burst stimulation dissociates attention and action updating in human inferior frontal cortex. Proc. Natl. Acad. Sci. U.S.A. 107, 13966–13971. 10.1073/pnas.1001957107 20631303 PMC2922216

[B26] Wessel J. R. Aron A. R. (2014). Inhibitory motor control based on complex stopping goals relies on the same brain network as simple stopping. Neuroimage 103C, 225–234. 10.1016/j.neuroimage.2014.09.048 25270603 PMC4396635

[B27] Wessel J. R. Conner C. R. Aron A. R. Tandon N. (2013). Chronometric electrical stimulation of right inferior frontal cortex increases motor braking. J. Neurosci. 33, 19611–19619. 10.1523/JNEUROSCI.3468-13.2013 24336725 PMC3858630

[B28] Whelan R. Conrod P. J. Poline J. B. Lourdusamy A. Banaschewski T. Barker G. J.. (2012). Adolescent impulsivity phenotypes characterized by distinct brain networks. Nat. Neurosci. 15, 920–925. 10.1038/nn.3092 22544311

[B29] Wiecki T. V. Frank M. J. (2013). A computational model of inhibitory control in frontal cortex and basal ganglia. Psychol. Rev. 120, 329–355. 10.1037/a0031542 23586447

